# Copland’s Dictionary of Practical Medicine

**Published:** 1845-08

**Authors:** 


*Copeland's Dictionary of Practical Medicine*, Edited by Professor Lee.

In our last number we stated that this publication had progressed as far as Part IX. Since then we have received all the numbers up to *Part X*, inclusive, but at an hour so late as to preclude us from much examination of their contents. The following subjects are treated of in *Part X*, viz :

*Gall-bladder and ducts ; Gangrene ; Gastro-enteric disease ; Glanders ; Gout ; Hæmorrhage ; Hæmorrhois ; Hair, alterations of ; Headache ; Hearing, loss of.*

A hasty glance has shown us that the work continues to be enriched by the labours of the Editor.

